# Application of Machine Learning Models for Tracking Participant Skills in Cognitive Training

**DOI:** 10.3389/fpsyg.2020.01532

**Published:** 2020-07-22

**Authors:** Sanjana Sandeep, Christian R. Shelton, Anja Pahor, Susanne M. Jaeggi, Aaron R. Seitz

**Affiliations:** ^1^Department of Computer Science, University of California, Riverside, Riverside, CA, United States; ^2^Brain Game Center, University of California, Riverside, Riverside, CA, United States; ^3^School of Education, University of California, Irvine, Irvine, CA, United States; ^4^Department of Psychology, University of California, Riverside, Riverside, CA, United States

**Keywords:** cognitive memory training, hidden Markov model, Bayesian filtering, video games, n-back training, deep-learning

## Abstract

A key need in cognitive training interventions is to personalize task difficulty to each user and to adapt this difficulty to continually apply appropriate challenges as users improve their skill to perform the tasks. Here we examine how Bayesian filtering approaches, such as hidden Markov models and Kalman filters, and deep-learning approaches, such as the long short-term memory (LSTM) model, may be useful methods to estimate user skill level and predict appropriate task challenges. A possible advantage of these models over commonly used adaptive methods, such as staircases or blockwise adjustment methods that are based only upon recent performance, is that Bayesian filtering and deep learning approaches can model the trajectory of user performance across multiple sessions and incorporate data from multiple users to optimize local estimates. As a proof of concept, we fit data from two large cohorts of undergraduate students performing WM training using an N-back task. Results show that all three models predict appropriate challenges for different users. However, the hidden Markov models were most accurate in predicting participants' performances as a function of provided challenges, and thus, they placed participants at appropriate future challenges. These data provide good support for the potential of machine learning approaches as appropriate methods to personalize task performance to users in tasks that require adaptively determined challenges.

## 1. Introduction

Over the last decade, the scientific topic of improving cognitive capacity by leveraging the plasticity of the brain has gathered both significant interest and controversies regarding effectiveness (Karbach and Unger, 2014; Au et al., 2016; Melby-Lervåg et al., 2016; Simons et al., 2016; Green et al., 2018; Soveri et al., 2018; Redick, 2019). For example, one of the most popular approaches is to train working memory (WM), a limited-capacity system involved in temporary storage and manipulation of information (Baddeley, 2012). Adaptive and extended WM training often improves WM skill (Soveri et al., 2017); however, the extent to which this type of training produces improvements that generalize far beyond the training task remains controversial (Melby-Lervåg and Hulme, 2013; Melby-Lervåg et al., 2016). A potential reason for inconsistencies across studies might be due to the fact that there are substantial individual differences in training outcomes; this might be because training trajectories are very diverse across participants, giving rise to the possibility that standard adaptive procedures may not provide the optimal challenge to all participants. For example, the N-back task (Pergher et al., 2019), which we study here, has been used widely to ameliorate cognitive declines in populations ranging from children with ADHD (Rutledge et al., 2012) to older adults with cognitive declines (Stepankova Georgi et al., 2013), however performance on the N-back ranges largely both within and across studies, as do the methods of adaptively adjusting task challenges to participants' abilities (Pergher et al., 2019). For effective cognitive training, we need better systems that can effectively estimate participants' capacity limits and provide appropriate challenges near those limits (Deveau et al., 2015; Pedullàa et al., 2016). A step in the right direction is to develop a system that could predict a participant's performance and use this information to determine the next challenge, with an overall goal to improve the participant's cognitive functions.

In the current project, we investigate the use of machine learning (ML) techniques to estimate participants' cognitive abilities during WM training and explore whether they could provide more optimal training experiences by more effectively aligning training challenges with participants' needs. We note that the current manuscript is restricted to addressing the skill of ML models to predict performance *during* training and we do not address issues of transfer. We employ a data-driven approach that examines data collected during training of a large number of participants and uses these sophisticated analysis tools to achieve better estimates of the appropriate challenge needed at each time for each participant. The key motivation of this approach is the observation that the behavior of a participant at a certain point of training not only depends on the momentary challenge, but also on the history of challenges and performance across these challenges. However, a limitation of commonly used adaptive procedures is that they typically adjust challenges only based upon the most recent information (e.g., a 3/1 staircase approach only looks at the last few trials and a block-wise approach only looks at the most recent block of trials). Thus, a participant with a few lapses (or lucky guesses) can improperly be set far back (or forward) in challenge level, which might be detrimental for participants' motivation and/or learning.

A potential solution to overcome such problems are statistical models that can temporally encode a participant's momentary skill and predict their performance for future challenges. Hidden Markov models (HMMs) are an appealing solution for such problems where the dynamics of observations are too complex to be directly modeled and are approximated via a discrete-state hidden Markov process. We analyze data gathered from WM training studies that use a variety of training approaches including different adaptive procedures across a large number of participants, together allowing for a broad sampling of challenges and accompanying levels of performance across the training experience. Our contribution is an algorithm that predicts a participant's performance at each time-point during training. We compare the proposed HMMs with other filtering (the Kalman filter) and deep-learning (LSTM) models, both of which were attempted, but produced poorer fits than the HMM model. Our approach sets the ground for future research that can evaluate the extent to which the use of these models to dictate challenges to the user (beyond the scope of the present manuscript) would provide a better training experience and/or benefits to training outcomes.

## 2. Materials

### 2.1. Training Programs

**Figure 1:**
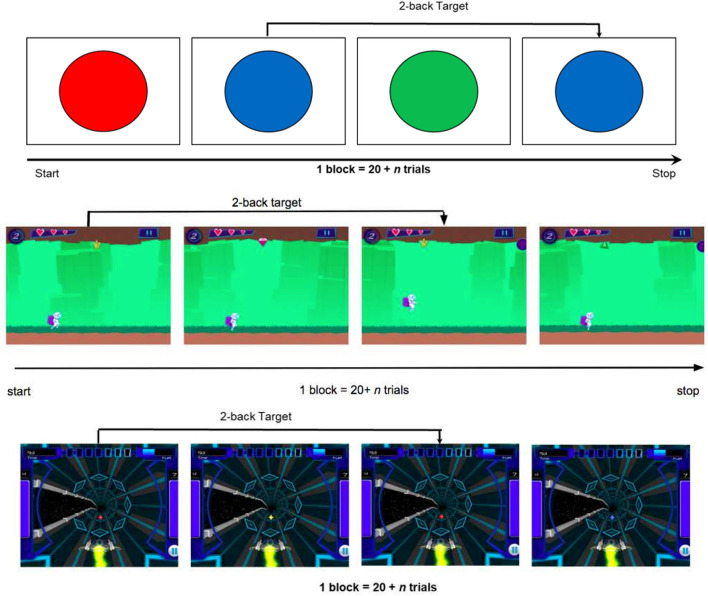
**(Top)** Example for a two-back level in the non-gamified Tapback condition. **(Middle)** Example for a two-back level in the gamified Recollect condition. **(Bottom)** Example for a two-back level in the gamified Recall condition (Mohammed et al., 2017).

The data sets were generated via N-back training software developed at the University of California Riverside Brain Game Center (Deveau et al., 2015). “Recall” and “Recollect the Study” are software applications that were used to collect data in Experiments 1 and 2, respectively. Both applications are available as free downloads on the Apple App store for iOS and Recollect is also available on Google Play. In both games, participants are presented with consecutive streams of stimuli of different shapes and colors, and the objective is to respond to stimuli that match those presented exactly N items earlier. For example in 1-Back, participants match the current item with the one just seen, and in 2-Back, they match the current item with the one presented two trials ago. Higher levels of N increase WM load and make the task more difficult. In these training programs, task difficulty is adaptively adjusted based on performance. The main difference between the two training programs is the game environment: Recall is a 3D space-themed game (video demo can be found here: https://www.youtube.com/watch?v=zx_t6paHB3Y) in which the participant is in control of a spaceship (Deveau et al., 2015; Mohammed et al., 2017). The participant must zap target stimuli while also collecting fuel pods for the space ship (non-targets). In contrast, Recollect is a platform game (video demo can be found here: https://www.youtube.com/watch?v=bUNrFk3eA3M) in which the participant is in control of an astronaut that must collect resources needed to feed a colony and keep the technology working (target stimuli), but must avoid non-targets and other obstacles. Both Recall and Recollect were contrasted against Tapback, a non-gamified N-back training program in which a series of colored circles is presented on a plain background and the participant must tap on the circles that are the same color as those presented N items earlier (see Figure 1). In all training paradigms, the stimuli are presented for 3 s, 30% of the stimuli are targets, and performance feedback is presented on every trial in the form of tones indicating correct or incorrect responses. Here we present data from two experiments, which differ both with respect to condition (gamified or non-gamified) and also in task-challenge progression (algorithm). Namely, in Experiment 1 a single adaptive algorithm was used across Recall and Tapback conditions, therefore we fit models on Recall and created the test-pool on Tapback. Experiment 2 featured a multitude of game-play-progression algorithms across Recollect and Tapback conditions. The training set consisted of Recollect and Tapback while the test set was on Tapback.

### 2.2. Participants

Two hundred and sixty-two participants at the University of California Riverside (UCR), or University of California Irvine (UCI), trained for 16–20 N-back training *sessions* of 20 min each, with 2 sessions conducted per day with a 10 min break between sequential sessions. Each session is broken down into multiple *blocks*, where a *block* corresponds to a 2–3 min period of continuous play. The two training studies took place between Fall 2014 and Fall 2016 (Experiment 1) and between Winter 2017 and Spring 2018 (Experiment 2). In the former, the sample consisted of 85 participants (mean age = 19.87 years, SD = 2.32; Nfemale = 41, Nmale = 35, Nother/Unknown = 7) randomly assigned to Recall (*N* = 49) or Tapback (*N* = 36) training. In the latter, 177 participants (mean age = 19.79 years, SD = 1.87; Nfemale = 93, Nmale = 76, Nother/Unknown = 8) were randomly assigned to Recollect (*N* = 85) or Tapback (*N* = 92) training. Participants provided written informed consent and received monetary compensation for participation. All studies were approved by the UCR and UCI Institutional Review Boards.

## 3. Methods

### 3.1. Filtering

We apply filtering approaches, common in statistics and signal processing to track an unknown quantity as it changes based on noisy measurements, as an appropriate framework for N-back training where the unknown quantity is participants' cognitive skill related to the task at hand (not to be confused with constructs such as general intelligence or even WM capacity) and the measurements are the participants' performances in the game. Here we review the notation and ideas behind such tracking, particularly as they relate to the solutions we employed. We refer to the work of Särkkä (2013) as a good practical introduction to the topic with more detailed information.

#### 3.1.1. Notation

We let *x*_*t*_ be the hidden unknown quantity at time discrete time step *t*. We let *y*_*t*_ be the observation made of *x*_*t*_ at the same time. One or both can be discrete or continuous, single-valued or vector-valued. As a statistical model, all of the variables (*x*_1_, *x*_2_, …, *y*_1_, *y*_2_, …) are probabilistically related to each other. In our case, *x*_*t*_ represents the (unknown, hidden) cognitive skill of the participant at time *t* and *y*_*t*_ is the performance of the participant at time *t*.

We consider **Markov** models (Chan et al., 2012) in which *x*_*t*_ captures all of the *state* of the system necessary for the future. That is, the past states, {*x*_1_, *x*_2_, …, *x*_*t*−1_}, are statistically independent of the future states, {*x*_*t*+1_, *x*_*t*+2_, …}, given the present state, *x*_*t*_. Similarly, the observation at time *t* depends directly only on the state at time *t* (that is, *y*_*t*_ is independent of everything else, given *x*_*t*_). Markov models are specified by two distributions: the transition distribution of *p*(*x*_*t*_ ∣ *x*_*t*−1_) that specifies the probability of any particular one-step state change, and the observation distribution of *p*(*y*_*t*_ ∣ *x*_*t*_) that specifies the probability of any particular observation given the current state.

The goal of filtering is to calculate the distribution of *x*_*t*_, given all of the evidence up to this point: *p*(*x*_*t*_ ∣ *y*_1_, *y*_2_, …, *y*_*t*_). This completely captures the information we currently have about the value of *x*_*t*_. In our case, this is the posterior distribution over the participant's cognitive skill, conditioned on the evidence up to this point. As *y*_1_, *y*_2_, …, *y*_*t*_ are observed and known, this is a function of *x*_*t*_, specifying the probability of a particular state, given the evidence. It is known as the *belief state*.

### 3.2. Modeling With a HMM

Hidden Markov models (HMMs) have been well validated as an effective temporal pattern recognition tool (Rabiner, 1990). Little to no work has previously reported on the use of HMMs to study psychological experiments of learning (Valsiner et al., 2009). However, based upon success of these models in similar domains, we hypothesize that HMMs are *powerful* models for representing dynamic changes in participants' training performances and for representing latent changes in the cognitive levels with concise state-transition paths. The Recall and Recollect datasets are well suited for an HMM architecture. We consider each participant as an example for the model. Time is quantified in units of *blocks*, each with a constant n-level difficulty. For a participant, the presented n-level signal (difficulty) has a dominating effect on the performance signal. Since participant performances can exhibit abrupt changes that are caused by external factors, the mapping between presented n-level and performance is not smooth.

**Figure 2:**
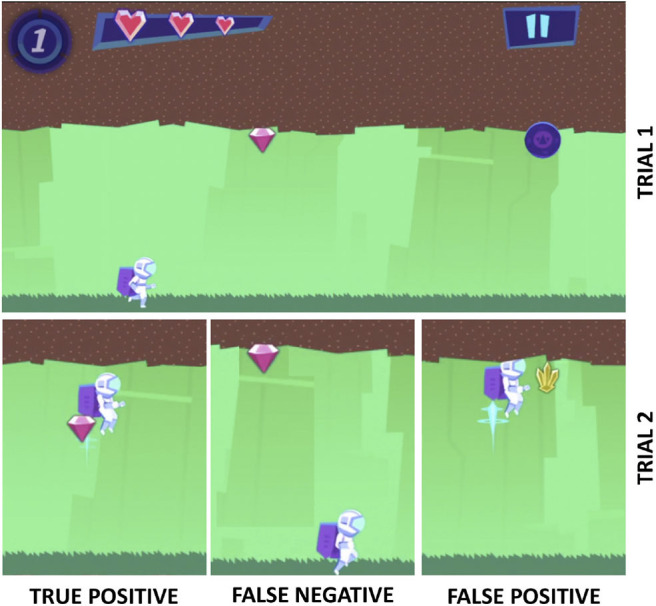
A visual of how the Recollect game translates to *accBlock*. It shows the 1-back task in which the player needs to collect items that match those seen 1 trial ago. The top part shows the first item in a 1-back task, a pink diamond (trial 1). The bottom part shows examples of responses in the next trial: the player either collects the target (also a pink diamond; true positive), or misses a target (false negative), or collects a non-target (yellow gem; false positive).

We introduce a metric for participant performance called *accBlock*. Figure 2 gives examples of the *true positives* (TP; collecting a target), *false negatives* (FN; missing a target), and *false positives* (FP; collecting a non-target) in context of the Recollect game. For all our experiments, accBlock is calculated in Equation (1).

(1) $$\begin{array}{r} accBlock=\frac{\Sigma_{block}TP}{\Sigma_{block}\left(TP+FN+FP\right)} \end{array}$$

**Figure 3:**
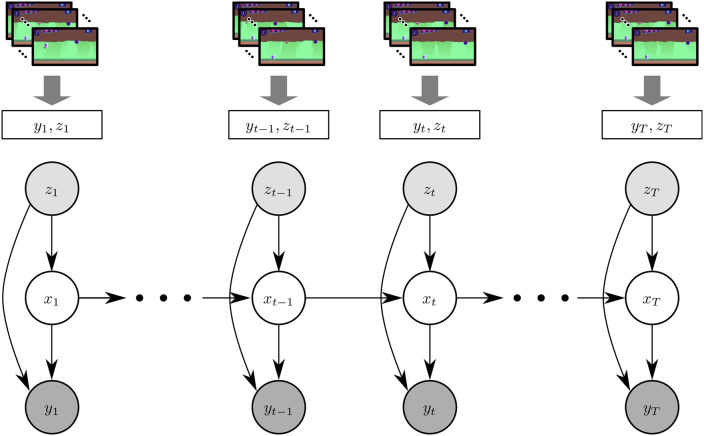
Graphical representation of the system. A block's value corresponds to a single node in the model. This is shown in the top portion of the figure, where *y*_*t*_, the accuracy achieved at time t, and *z*_*t*_, the presented n-level of the task at time t, are for a single block and that translates into a node in the model. The bottom is the graphical representation of the hidden Markov model. The arrows represent direct conditional dependencies among the modeled variables. The state transition model (from *x*_*t*−1_ to *x*_*t*_) is described in section 3.2.3.

In order to adapt the HMM to a cognitive training context, we assume that (1) block-accuracy is generated by a latent state, and (2) the state at time *t* is conditioned on the local history *t* − 1 (Rabiner, 1990). Formally, this these correspond to the emission and a transition distributions of an HMM. The presented n-level signal acts as a driving input, making it an *input-output HMM* (IOHMM) (Bengio and Frasconi, 1994), whose dependency diagram is shown in Figure 3.

We use the following notations.

- *x*_*t*_, the latent cognitive skill, is the participant's maximal n-level skill at time t, *x*_*t*_ ∈ {1, 2, … *N*}.
- *y*_*t*_ is the accuracy achieved at time t.
- *z*_*t*_ is the presented n-level of the task at time t.
- *m*_*t*_ is the number of targets during the block at time t.
- *t* is the index of the training *block*, *t* ∈ {1, 2, … *T*}.
- A participant (an instance or training sequence) is denoted by *i*. The above quantities are also subscripted with *i* when necessary to distinguish between their values per participant.

Our filtered belief state over *x*_*i,t*_ is our estimate of the maximal N-back skill of participant *i* at time *t*.

#### 3.2.1. Accounting for Heterogeneity

Participants' performances are affected not only by their own cognitive skills, but also by the difficulty of the task presented. To incorporate this information, we use item response theory (IRT), (Rasch, 1980) a modeling paradigm frequently used in educational testing. Within commonly used IRT, probabilistic models are used to describe the relationship between observable item responses and unobservable psychological abilities (Hambleton and Swaminathan, 1984). The strength of IRT models lie in separating the effects of individuals and specific items. The basic assumptions are as follows. (1) The probability that a participant will score correctly on an item (in a trial) follows a specific parametric functional form called the item characteristic curve (ICC), which depends on parameter(s) for that participant and the item. (2) The items are dichotomous, and the ICC is strictly monotonic on the latent trait scale. And, (3) given the participant's skill, the items are considered conditionally independent. Countless extensions and generalizations of the IRT model have been developed, including modeling attitudinal changes with state space models (Martin and Quinn, 2002) and modeling growth in reading skill with state space models (Wang et al., 2013). Notable extensions of IRT that make it more amenable to our datasets are adaptive item administration, and time-varying abilities. The dynamic IRT equation we use in constructing the observation (emission) probabilities is given by the four-parameter logistic (4PL) IRT model (Barton and Lord, 1981):

(2) $$\begin{array}{r} a_{i,t}=c_{i}+\frac{\left(d_{i}-c_{i}\right)}{1+e^{-\rho_{i}\left(x_{i,t}-z_{i,t}\right)}} \end{array}$$

where at block *t*, *x*_*i,t*_ is the skill of the participant *i*; the difficulty of a test item is given by *z*_*i,t*_ which corresponds to the presented n-level; ρ_*i*_, *c*_*i*_, and *d*_*i*_ are static item parameters accounting for the participant's discrimination, guessing skill and carelessness (noise), respectively; and *a*_*i,t*_ is the Bernoulli item response variable that corresponds to the correctness of the response to a stimulus in an block.

#### 3.2.2. Emission Architecture

As mentioned earlier, each stimulus or trial in a block can either be a correct or an incorrect response and is described by a *Bernoulli* random variable, whose parameter (probability of correct response) is given in Equation (2). This IRT model assumes that the accuracy of participant *i* at block *t*, *a*_*i,t*_ is driven solely by the participant's skill at time *t*, *x*_*i,t*_ and the difficulty of the presented items, *z*_*i,t*_. This results in a Binomial distribution for the overall accuracy^1^ of the participant during the block:

(3) $$\begin{array}{r} y_{i,t}|\left(x_{i,t},z_{i,t},m_{i,t}\right)\sim \text{Bin}\left(m_{t},{â}_{i,t}\right) \end{array}$$

where â_*i,t*_ would be the same as the *a*_*i,t*_ from Equation 2.

However, we acknowledge that there are between-block variations (in attention or fatigue, for instance) that necessitate dependence among responses within a block, even after conditioning on the participant's skill and the task difficulty. Thus, we model â_*i,t*_ as not exactly equal to *a*_*i,t*_ (but still a single value, shared across all responses in the block). This new random variable, â_*i,t*_, can be thought of as the per-block realization of the participant's skill, and it serves to couple the (otherwise independent) responses within the block. We let â_*i,t*_ be a random variable drawn from a Beta distribution, with mean of *a*_*i,t*_:

(4) $$\begin{array}{r} {â}_{i,t}\sim \text{Beta}\left(\alpha a_{i,t},\alpha \left(1-a_{i,t}\right)\right) \end{array}$$

where α controls the concentration of the distribution around *a*_*i,t*_. In this new model, to get the probability of the observation *y*_*i,t*_, given the presented n-level, *z*_*i,t*_ and any postulated latent skill, *x*_*i,t*_, we marginalize over possible realized abilities (skipping the straight-forward mathematical derivation):

(5a) $$\begin{array}{r} P(y_{i,t}|x_{i,t},z_{i,t},m_{i,t})=\int_{0}^{1}P\left(y_{i,t}|{â}_{i,t},m_{i,t}\right)P\left({â}_{i,t}|x_{i,t},z_{i,t},m_{i,t}\right)d{â}_{i,t} \end{array}$$

(5b) $$\begin{array}{r} =\frac{\Gamma \left(\alpha \right)\Gamma \left(m_{i,t}+1\right)}{\Gamma \left(\alpha a_{i,t}\right)\Gamma \left(\alpha \left(1-a_{i,t}\right)\right)\Gamma \left(a_{i,t}+m_{i,t}\right)}\\ \times \frac{\Gamma \left(y_{i,t}+\alpha a_{i,t}\right)\Gamma \left(m_{i,t}-y_{i,t}+\alpha \left(1-a_{i,t}\right)\right)}{\Gamma \left(y_{i,t}+1\right)\Gamma \left(m_{i,t}-y_{i,t}+1\right)} \end{array}$$

While complex looking, it is simple for a computer to evaluate using standard library functions for the gamma function (a generalization of factorial).

#### 3.2.3. Transition Architecture

The transitions between the states are modeled as a Markov process. The *N* × *N* transition matrix for our system specifies the probability of the latent state transitioning from *x*_*t*−1_ to *x*_*t*_:

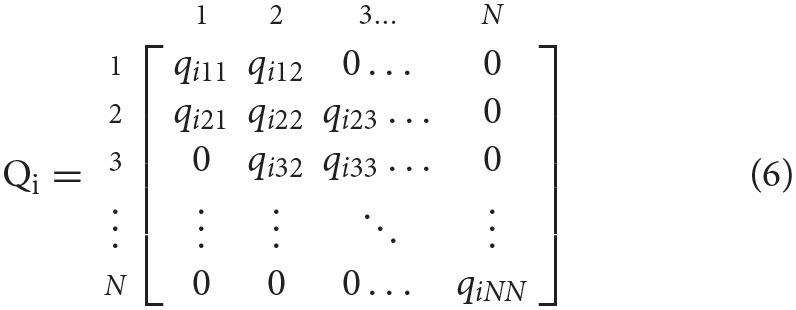



where $q_{i,x,x^{\prime }}$ is the probability that participant *i*'s hidden state will transition from *x*_*t*−1_ = *x* to $x_{t}=x^{\prime }$ given that he or she experienced n-level *z* and performed as *y* at *t* − 1. In applying this model in the context of n-level relationships, we put a structure on the general transition matrix. Transitions are only allowed between adjacent neighbors. In the context of our application, this assumption is both behaviorally and empirically grounded. Since consecutive hidden n-level skill jumps are at most 1, we are forcing a smoothness to the progression (or degradation) of a participant's skill.

In **model-1**, we assume that this probability does not depend on *y* or *z*:

(7) $$\begin{array}{r} q_{i,x,x^{\prime }}^{\left(y,z\right)}=q_{i,x,x^{\prime }}=\{\begin{array}{cc} 0, & \text{if}|x-x^{\prime }|>1\\ q_{x-x^{\prime }} & \text{otherwise} \end{array} \end{array}$$

That is, we just have just 3 parameters: *q*_−1_, *q*_0_, *q*_+1_, the probabilities that the participant's latent skill will transition down one level, stay the same, or transition up one level^2^.

While this assumption reduces the number of parameters, it fails to capture important cognitive behavior causing change in n-levels (such behavior is viewed as just part of the noise). As such, we introduce heterogeneity into the transitions. In **model-2**, we assume that a participant's latent skill's propensity for transitioning between n-levels is affected by the previous observed accuracy, and the previous n-level challenge presented. For example, a participant at n-level 5 and a block-accuracy of 0.9 has a higher chance of transitioning to a level-6, when compared to a block-accuracy of 0.5. We note here that we are describing the models' estimate as the participants actual challenge in these data sets depends upon the adaptive algorithms used in the training procedure. Hence, the transition probabilities are conditional on external variables besides *x*_*t*−1_. We model the effects of two features on the transition probability, (1) the difference between the predicted n-level and the presented n-level at the previous time-step and (2) the observed block-accuracy from the previous time-step.

We discretize the observed accuracy, *y* into 10 evenly spaced bins. Let *B*(*y*) be the discrete bin into which *y* falls. Our form for the above matrix elements is

(8) $$\begin{array}{r} q_{i,x,x^{\prime }}^{\left(y,z\right)}=\{\begin{array}{cc} 0 & \text{if}|x-x^{\prime }|>1\text{or}|z-x^{\prime }|>2\\ q_{\left(x-x^{\prime }\right),\left(z-x^{\prime }\right),B\left(y\right)} & \text{otherwise} \end{array} \end{array}$$

**Figure 4:**
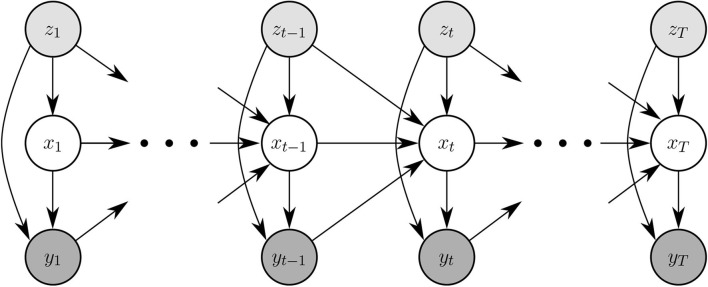
Graphical representation of a history-driven HMM. Generation of *y*_*t*_, *z*_*t*_ from a block remains the same as in Figure 3.

Thus, we now have 3 × 5 × 10 = 150 parameters: 3 transitions in the participant's latent skill (*x*−*x*′ ∈ {−1, 0, +1}) for 5 differences in presented n-level and participant's latent skill (*z* − *x*′ ∈ {−2, −1, 0, 1, 2}) and 10 different performance levels. Figure 4 shows state transitions that are additionally conditioned upon the previous block-accuracy and presented n-level. In both models, we also have parameters specifying the starting distribution of the participant's latent skill.

#### 3.2.4. Training the HMM

To infer the most likely parameters of our model given an observed sequence of block-accuracies, we adapt the standard Baum–Welch expectation-maximization (EM) algorithm (Baum et al., 1970; Dempster et al., 1977) to stimulus-dependent emission and transition densities. The crucial differences are that (1) the emission probabilities are fixed through IRT, and (2) many of the parameters in the transition matrix (Equation 6) are the same leading to tied parameters. Both are standard extension in HMM training. In our settings, the convergence criteria is when the slope of the log-likelihood function falls below a threshold value. We choose this value to be 0.001 (to allow for a little imprecision). The IRT observation model parameters, (ρ; *c*; *d*; α) are required in Equation (2). Since, there are only a few of them to explore, we optimize them in a grid-search loop (outside of the Baum-Welch algorithm).

## 4. Results

In the first experiment, both Recall and Tapback utilized the same game-play-progression algorithm, therefore we fit the models on the Recall dataset, and create the test-pool with the Tapback dataset. In Experiment 2, a multitude of game-play-progression algorithms was used across Recollect and Tapback conditions hence we fit the models and evaluated performance by a 80:20 split.

### 4.1. Experiment 1

The purpose of this experimental setup is to investigate the generalization power of the models across different game environments and settings using Tapback and Recall. Both training paradigms contained a 4-item stimulus set, although only Recall was designed to engage multiple sensory systems. Specifically, in Tapback the participant was presented with 4 colors whereas in Recall each of the colors was paired with a unique shape and sound. N-level progression was adjusted adaptively using the same algorithm in the two training paradigms: consistent accuracy above 85% in a block led to an advancement of N-level and consistent accuracy below 70% in a block led to a decrement of N-level.

**Figure 5:**
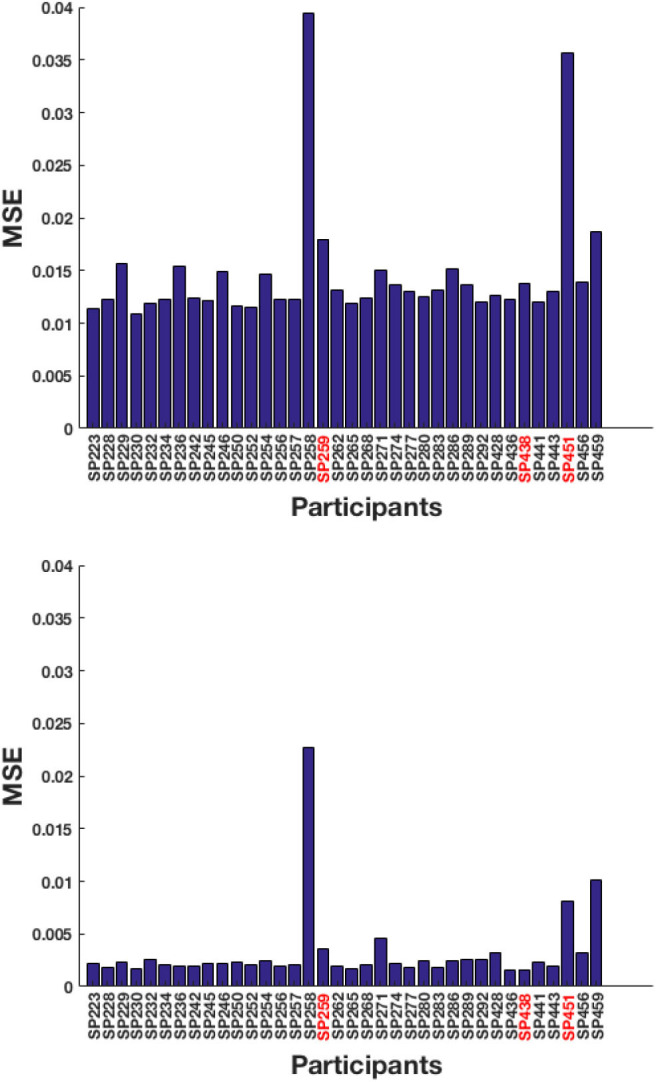
Test MSE across all participants for **(top)** model-1 and **(bottom)** model-2. The participants highlighted in red correspond to the samples picked below for analysis.

A total of 49 training sequences from Recall are used and the test-pool consists of 36 sequences from Tapback. At a behavioral level, the two groups of participants did not differ in mean N-level achieved in the last training session (Recall: *M* = 3.38, SD = 1.19; Tapback: *M* = 3.65, SD = 1.56). The overall model performance captured by test RMSE, for model-1 and model-2 are 12.06 and 5.60%, respectively, showing that model-2 (history driven hmm) is a better fit when compared to model-1 (hmm with a universal transition matrix). The MSE across all participants for model-1 and model-2 are shown in Figure 5. The universal transition matrix for model-1 is shown in 4.1. An extremely high proportion of weights on the main diagonal, indicates a model that is insensitive to small changes, and is likely to maintain a participant at consistent n-levels. Since model-2 has transition matrices that vary across each time-step and for each participant The non-homogeneous transition matrices for each sample participant for selective time-steps are shown separately in the Supplementary Material section. The hyper-parameters (IRT observation model parameters) (ρ; *c*; *d*; α) are (1.6;0;1;11.56).

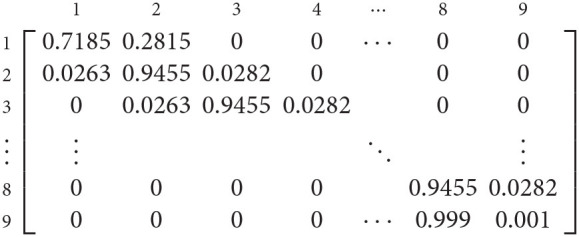



Next, we detail model behavior for some selected samples from the test dataset to help illustrate how both models fit individual participants. SP259, SP438, and SP451 are the chosen examples, since their N-level trajectories are illustrative of the variation that can be found within the dataset.

#### 4.1.1. SP438

**Figure 6:**
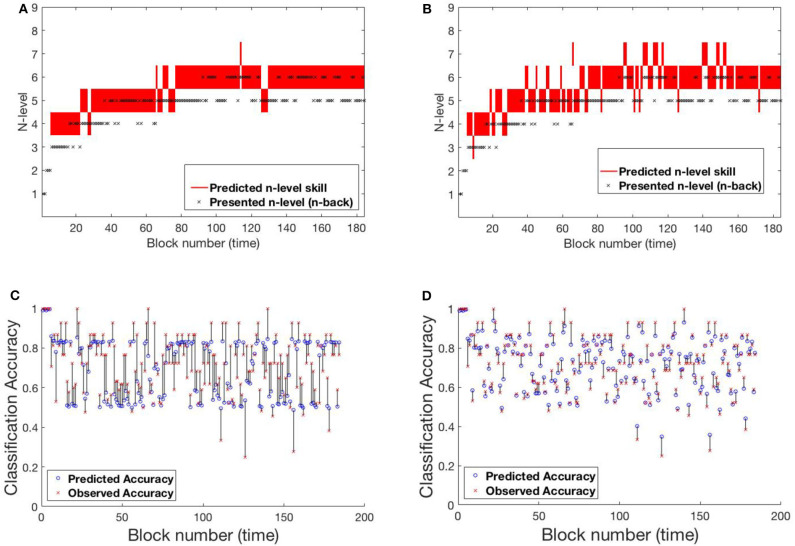
Comparing models for participant SP438. **(A)** Trajectory of estimated WM skill from model-1. **(B)** Trajectory of estimated WM skill from model-2. **(C)** Prediction vs. observed block accuracy at each block from model-1. **(D)** Prediction vs. observed block accuracy at each block from model-2.

Figure 6 shows participant SP438 performance in the Tapback condition. The participant practices numerous blocks of trials where n-level adapts in a blockwise fashion and improvements in estimated WM skill is steady and at a typical rate, progressing from 4 to 5 to 6 across training. N-level is adjusted when performance is above .8 or below .6 within a given block. Since accuracies for most blocks are moderate, the presented n-levels only change occasionally. Both models adjust their estimate of the participant's WM skill before the actual increase in presented n-levels happens. This behavior is seen at block 30 and block 80. Figure S1 shows the evolving transition matrix for model-2 at this change. Model-1 maintains the cognitive load of the participant between level 4 and 6 with a single rise to 7. The trajectory from model-2 is less smooth with a higher tendency to promote, and then demote, the participant.

#### 4.1.2. SP259

**Figure 7:**
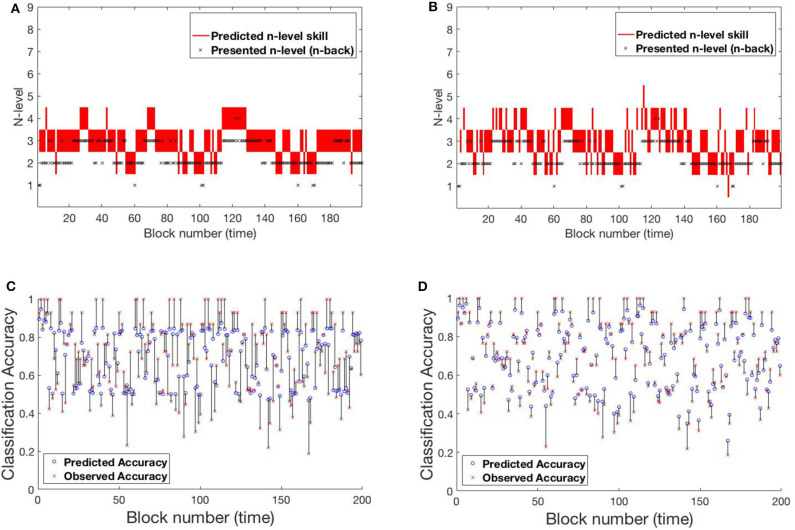
Comparing models for participant SP259. **(A)** Trajectory of estimated WM skill from model-1. **(B)** Trajectory of estimated WM skill from model-2. **(C)** Prediction vs. observed block accuracy at each block from model-1. **(D)** Prediction vs. observed block accuracy at each block from model-2.

Participant SP259 (Figure 7) shows a very different performance profile, which is typical of someone who did not show learning on the task. We note that participants like this are very common in n-back training studies and that addressing their data is both important to ensure that their data can be accounted for by the models and that understanding their performance may provide avenues toward better catering to their needs in the future. Their assigned n-level vacillates mostly between n-level 2 and 3 with occasional episodes at 1 or 4. Further, even at n-level of 2 accuracy varies over a wide range (0.2–1.0), suggesting that this participant may not have been consistently engaged in the task. We see that model 2, again, more closely follows the momentary performance of the participant, with promotions and demotions being prominent in the trajectory of estimated WM skill. For example, after the participant completes playing a level-4 trial with poor accuracy at block 124, model-2 demotes him/her to level 3, while model-1 retains him/her at level 4 for a few more blocks. Figure S2 shows the evolving transition matrix for model-2 during this demotion. We note that the WM skill for this participant, would be difficult to estimate for any model. Clearly, the participant did not consistently perform at their skill. With the data provided, it is difficult to estimate the difference between a lapse in performance and a true change in WM skill.

#### 4.1.3. SP451

**Figure 8:**
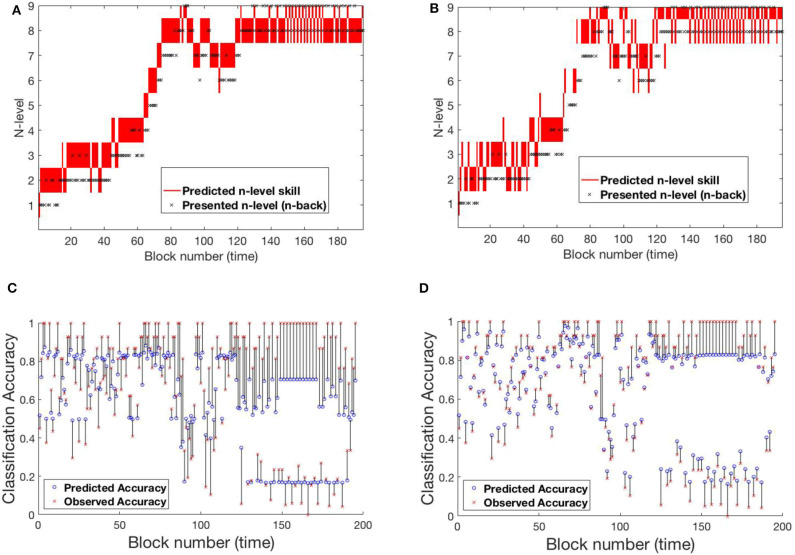
Comparing models for participant SP451. **(A)** Trajectory of estimated WM skill from model-1. **(B)** Trajectory of estimated WM skill from model-2. **(C)** Prediction vs. observed block accuracy at each block from model-1. **(D)** Prediction vs. observed block accuracy at each block from model-2.

A 3rd pattern of performance is found in SP451 Figure 8, who showed substantial improvements on the trained task, but with occasional set-backs and oscillations in n-level patterns at an asymptote at n-level 8. To compare the performance of the models, we focus on the specific sequence of patterns from block 120 to block 195, where they performed most differently from each-other. Notice that the participant's performance is unstable between levels 8 and 9 and the underlying training algorithm throws him/her back and forth based on performance. Low and high accuracy levels correspond to presented n-levels 9 and 8, respectively. In this circumstance, model-1 is more likely to estimate WM skill at 8 (higher weights on the main diagonal of the transition matrix), while model-2 estimates WM skill at 9 (effect of accuracy at the previous time-step on transitions). Figure S3 shows model-2's evolving transition matrix for a n-level sequence 9-8-9. Here we note that the participant's true WM skill is most likely somewhere between 8 and 9, where they could sometimes perform well at 9 but couldn't maintain good performance at that level.

### 4.2. Experiment 2

Unlike Experiment 1, where the underlying dataset consisted of only one game-play-progression algorithm, Experiment 2 consists of Recollect and Tapback datasets, which utilized multiple game-play-progression algorithms. Both training paradigms contained an 8-item stimulus set, although only Recollect was designed to engage multiple sensory systems (each color was paired with a unique shape and sound). N-level progression was adjusted adaptively using a multitude of algorithms (see Table 1). The training set consisted of 142 Recollect and Tapback sequences whereas the test set consisted of 35 Tapback sequences. The two sets did not differ in mean N-level achieved in the last training session (Training set: *M* = 4.03, SD = 1.50; Test set: *M* = 4.13, SD = 1.43). In Table 1, errors consists of FNs and FPs.

**Table 1:** Game-play progression algorithms used in Experiment 2.

**Algorithm**	**Level up**	**Level down**	**Structure**
Staircase classic	3 hits	2 errors	Within blocks
Staircase moderate	3 hits	3 errors	Within blocks
Staircase difficult	6 hits	2 errors	Within blocks
Mini-block moderate	<6 errors in 40-trial block	>8 errors in 40-trial block	Between blocks
Mini-block difficult	<3 errors in 40-trial block	>6 errors 40-trial block	Between blocks
Mini-block reset	<3 errors in 40-trial block	>6 errors 40-trial block	Starts session at 2-back

**Figure 9:**
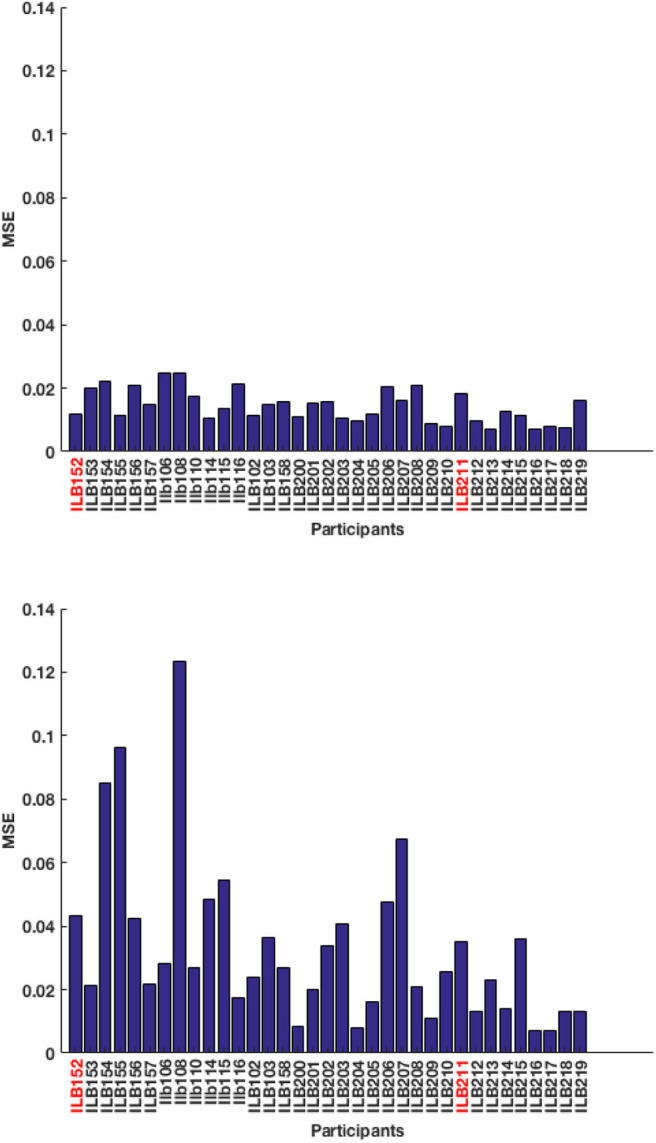
Test MSE across all participants for **(top)** model-1 and **(bottom)** model-2. The participants highlighted in red correspond to the samples picked below for analysis.

A few of these algorithms are more liberal in assigning participants with challenges that are inappropriate to their skill level, and where the models should show estimated WM skills that are different from presented n-level. The MSE across all participants for model-1 and model-2 are shown in Figure 9. The test RMSE of model-1 and model-2 are 12.54 and 18.52%, respectively. From these values, it can be inferred that both models are equally powerful in terms of performance. For this experiment, model-1 is a better fit. The reason for degraded performance for model-2 is as follows. Recollect is a high variance dataset that needs to train relatively more number of parameters for non-homogeneous state transitions. The number of training sequences used in Experiment 1 were 49 and were all of only one type of training algorithm. Even though the number of training sequences in Experiment 2 is 142, there are six different kinds of underlying training algorithms. Perhaps more data will aid in effective performance. We also surmise that the underlying training algorithms lead participants to inappropriate challenges, that causes an increased uncertainty associated with the previous tag (*x*_*t*−1_ − *z*_*t*−1_). As such, model-2 struggles with training (done with respect to the previous tags). The universal transition matrix for model-1 is shown in 9. Again, we see that higher ratio of weights on the main diagonal contributes to consistency. The most optimal hyper-parameters (IRT observation model parameters) obtained are (ρ; *c*; *d*; α) are (1.7;0;1;3).

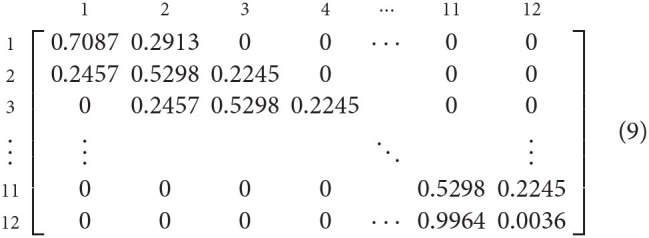



Now, we study the trajectories of specific samples from the test-pool to illustrate effectiveness of fits and draw comparisons between the models. RLB102 and RLB162 are the chosen participants for analysis.

#### 4.2.1. RLB102

**Figure 10:**
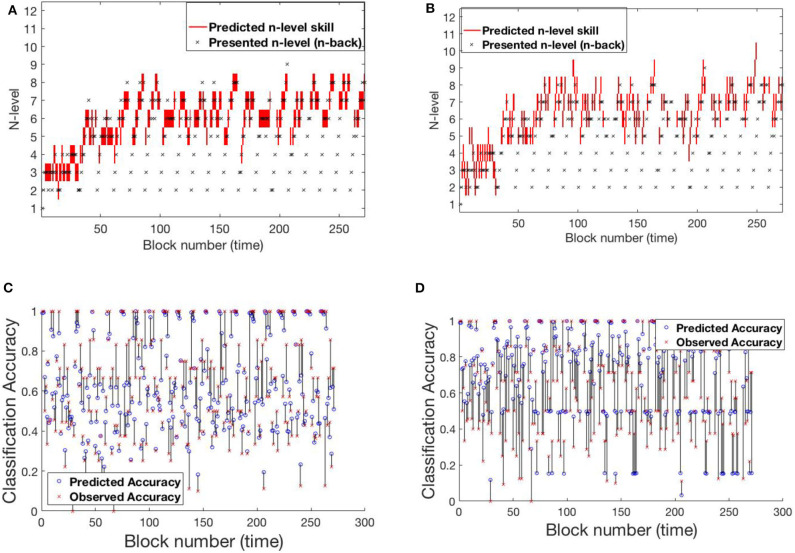
Comparing models for participant RLB102. **(A)** Trajectory of estimated WM skill from model-1. **(B)** Trajectory of estimated WM skill from model-2. **(C)** Prediction vs. observed block accuracy at each block from model-1. **(D)** Prediction vs. observed block accuracy at each block using model-2.

Figure 10 shows participant RLB102 from the Recollect dataset playing *Mini-block Reset*. Here, the program resets to n-level 2 at the start of every training session. This is an ideal setting to verify that our models do not simply trace trajectories that follow presented n-levels, but instead capture an estimate of participants' WM abilities. The estimated n-back skill level traces show that both models are successful in capturing cognitive levels that widely differ from presented n-levels at the times of reset. Model-1 has a smoother and more confident trajectory when compared to model-2. This behavior can be attributed to high weights on the main diagonal. However, both models oscillate a bit given the inconsistent performance of the participant.

#### 4.2.2. RLB162

**Figure 11:**
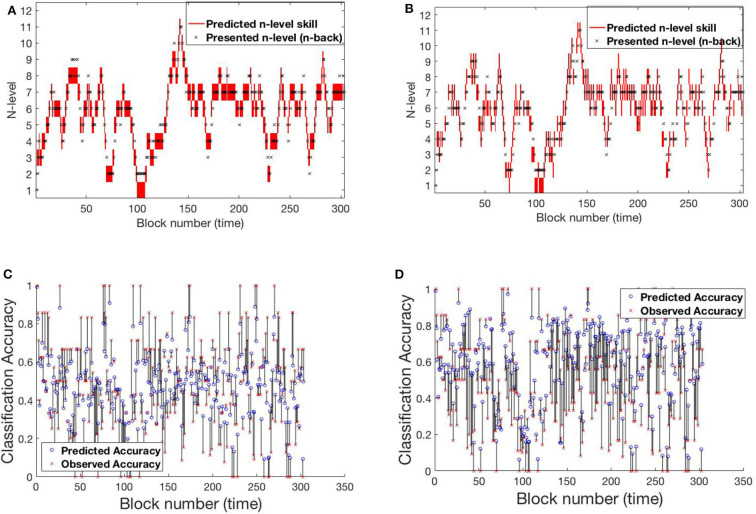
Comparing models for participant RLB162. **(A)** Trajectory of estimated WM skill from model-1. **(B)** Trajectory of estimated WM skill from model-2. **(C)** Prediction vs. observed block accuracy at each block from model-1. **(D)** Prediction vs. observed block accuracy at each block from model-2.

Figure 11 shows participant RLB162 from the Recollect dataset, who is in a condition called *Mini-block Moderate* where the n-level increases when performance is at a moderate level (e.g., 0.6 as opposed to 0.85 for most other algorithms). Both models represent substantial rises and falls in estimated WM skill, likely because this algorithm maintains performance at low accuracies, preventing a stable estimate of true skill. Nevertheless, model-1 does a better job of achieving relatively stable estimates at intermediate n-levels, and achieves greater confidence in these estimates, whereas model-2 follows experienced n-levels to a greater extent and exhibits little confidence in its estimates. This re-affirms the superior behavior of model-1 in keeping consistent n-levels. Figure S4 shows the evolving transitions from block 128 to 131. Across these blocks, the rise from n-level 5 to n-level 8 occurs in consecutive trials.

These sample participants provide a glimpse of how the HMMs are powerful in accurately predicting performances. While both models provide good fits, they differ in the extent to which they follow the momentary performance of different individuals, with model-1 providing relatively more stable estimates. While in Experiment 1, model-1 provides relatively poorer fits, and may be appropriate given the participants' true WM skills are not expected to change quickly. This may be more ideal for effective WM training, as an engaging algorithm must provide an appropriate challenge and avoid dropping or jumping based upon performance lapses or lucky streaks in performance.

To understand how other model frameworks would address the current dataset, we also fit an unscented Kalman filter (UKF) and a LSTM model.

#### 4.2.3. Kalman Filter Model

A Kalman filter (KF) is a special case of a Markov model where the states are continuous-valued (in contrast to the discrete-valued states of an HMM). The transition and observation models therefore operate on vectors. They are linear functions, with normally distributed, additive noise. They can be extended (at the cost of making calculations approximate) to a general non-linear observation function, **g**:

(10) $$\begin{array}{r} x_{t}=\text{A}x_{t-1}+\text{B}z_{t}+{\text{v}}_{t},\;{\text{v}}_{t}\sim N\left(0,Q\right) \end{array}$$

(11) $$\begin{array}{r} y_{t}=\text{g}\left(x_{t},u_{t},z_{t}\right)+{\text{w}}_{t},\;{\text{w}}_{t}\sim N\left(0,R\right) \end{array}$$

In our HMM models, we have only one item-parameter, ρ, for all participants. This assumes all participants' performance degrades (or improves) in the same way as the difficulty increases (or decreases) away from their maximum skill level. However, in a practical scenario this assumption might not be true, and we would like to model differences between participants and within a participant over time in ρ. With the Kalman filtering framework, we just add an extra dimension to the state variable to track the value of ρ. Thus, our KF model gains personalization at the cost of assuming linear dynamics in the estimated cognitive skill. The observation model (11) is chosen to be the IRT (Equation 2). Since, this is non-linear in nature, we use an unscented Kalman filter (UKF) for estimation (Julier and Uhlmann, 1997).

In Equation (10), **A** is the state transition matrix (2 × 2) applying the first-order Markov effect; **B** is the control input matrix (2 × 3) that applies the effect of the control input parameters on the states, the process noise for the parameters in the state space framework is **v**_*t*_, where it is assumed to be drawn from a zero mean multivariate normal distribution with covariance matrix **Q** (2 × 2). Equation (11) describes the measurements of the system. Function **g** maps the states and the control inputs to the observations. Similar to the process noise is the observation noise, **w**_*t*_, that is assumed to be zero mean Gaussian white noise, with covariance matrix **R** (1 × 1). Only the measurement sequence is observed while the state and the noise variables are latent. The parameters that are estimated via EM training are **A**, **B**, **Q**, **R**, **x**_0_ (initial mean of state variable), and **V**_0_ (initial variance of the state variable).

**Figure 12:**
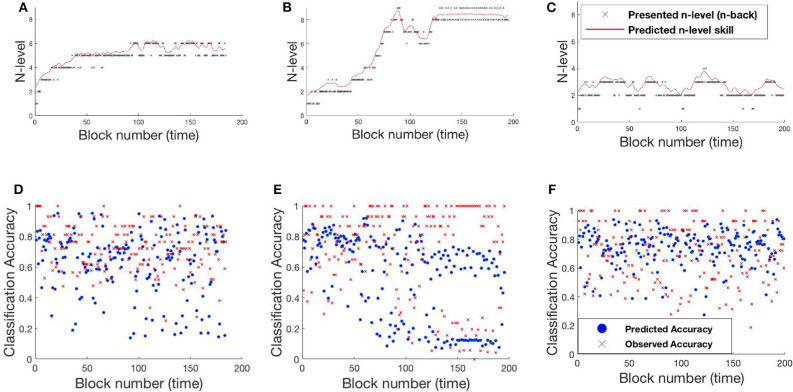
Samples from experiment 1. **(A,D)**: SP438, **(B,E)**: SP451, **(C,F)**: SP259. **(A–C)** Estimated n-level trajectory using UKF model. **(D–F)** Prediction vs. observed block accuracy at each block using UKF model.

**Figure 13:**
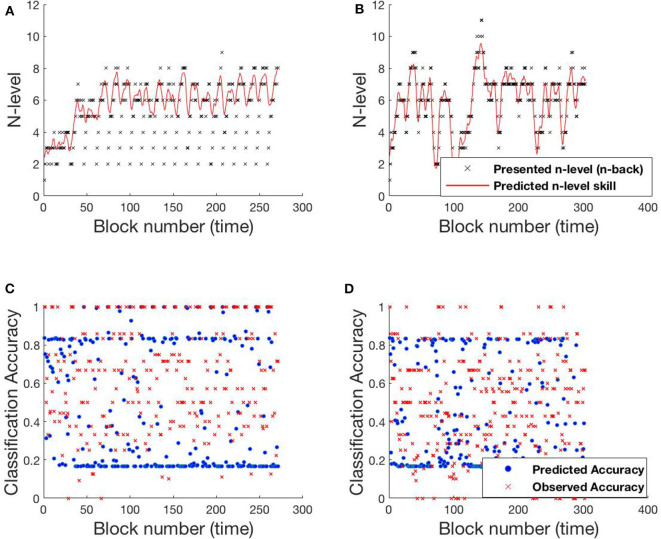
Samples from experiment 2. **(A,C)**: RLB102, **(B,D)**: RLB162. **(A,B)** Estimated n-level trajectory using UKF model. **(C,D)** Prediction vs. observed block accuracy at each block using UKF model.

The RMSE values for the UKF model are 18.83 and 31.52% for Experiment 1 and Experiment 2, respectively. From these values, it can be clearly seen that the UKF model performs poorly in both experiments when compared to the HMM. High error values in predictions for Experiment 2 re-affirms high variance in the underlying dataset. We show behavior of the UKF for the above chosen samples in Figures 12, 13. The predicted n-level curves are smooth and jump less often between consecutive n-levels. However, we see an increased error in predicting accuracy. For example, for SP451 (Figures 12B,E), the model appears to do a good job of estimating WM skill, with estimated memory scores consistently midway between levels 8 and 9. However, the gap between predicted and actual performance is large compared to that predicted by the HMM models. Further, for participant RLB102 (playing *mini-block moderate* game play progression training algorithm) in Figure 13 shows that the predicted n-levels appropriately vary significantly from the presented n-levels (desirable behavior similar to the HMMs). As such, we may conclude that despite the poor prediction of accuracy levels, this UKF model is successful at estimating participants' WM skill levels, and in fact is capable of making predictions that are intermediate between integer n-levels.

#### 4.2.4. LSTM Model

Deep neural networks have improved the state of the art for a wide variety of tasks, because of the availability of high computation power. Long Short Term Memory Recurrent Neural Networks (LSTMRNNs) (Hochreiter and Schmidhuber, 1997) are shown to be highly effective in sequence training tasks (Rebane, 2018). Their methods are similar to the filtering approaches above, but with important differences. Typically, the observed sequence {*y*_1_, *y*_2_, …} is linked through an unobserved hidden state sequence {*h*_1_, *h*_2_, …}. The process posits a function *f* that maps *h*_*t*−1_ and *y*_*t*−1_ to *h*_*t*_: *h*_*t*_ = *f*(*h*_*t*−1_, *y*_*t*−1_). There is a further observation function that maps *h*_*t*_ to *y*_*t*_. While *h*_*t*_ appears to mirror the *x*_*t*_ from the filtering framework above, critically, *f* (the transition mapping) is deterministic in most deep learning sequence frameworks. Furthermore, the past observation directly affects the transition of the hidden state. In such deep learning methods, *f* is usually chosen based on its computational and optimization properties and is often quite flexible (has many parameters). Note that when used, the hidden state *h*_*t*_ directly encodes all of the information about past observations. By contrast, in the filtering approach, the belief state takes the same role and thus the full distribution of *x*_*t*_ given the evidence (not *x*_*t*_ itself) is analogous to *h*_*t*_.

We also examined the extent to which a deep neural network, in this case an LSTM model, can fit our datasets. The input to the model is a 2D vector with the presented n-level *z*_*t*_, and the observed accuracy *y*_*t*_. The output is a 2D vector with the predicted n-level *x*_*t*_, and the predicted accuracy *y*_*t*_. The input and target tensors are processed collectively over all the samples during training. For the sequential model, the dataset is pre-processed as sliding windows, to effectively produce batches of timeseries inputs and targets. The model has an LSTM layer with 32 units (number of parameters: 4,480), on top of which lies a dense layer (number of parameters: 66) that converts the output into the desired 2D output. This configuration was chosen as the best by cross-validation.

**Figure 14:**
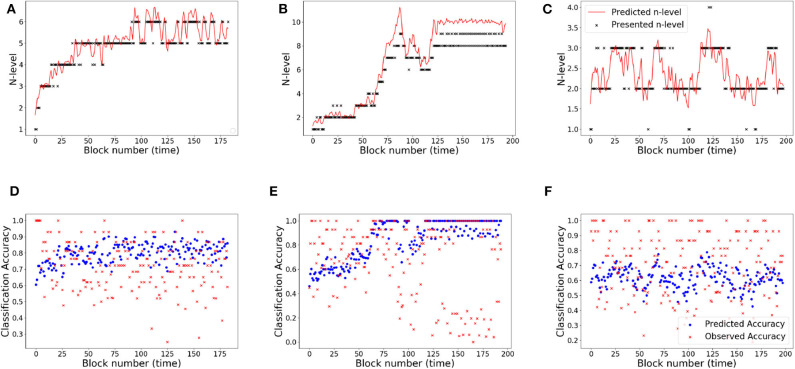
Samples from experiment 1. **(A,D)**: SP438, **(B,E)**: SP451, **(C,F)**: SP259. **(A–C)**: Estimated n-level trajectory using LSTM model. **(D–F)** Prediction vs. observed block accuracy at each block using LSTM model.

**Figure 15:**
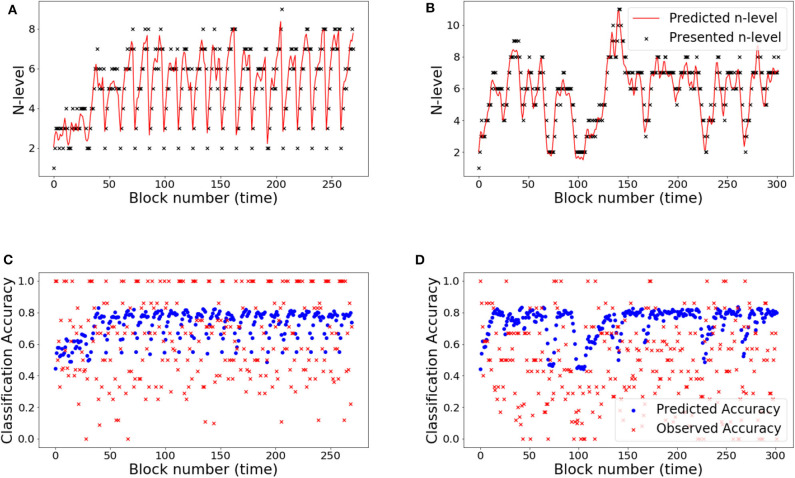
Samples from experiment 2. **(A,C)**: RLB102, **(B,D)**: RLB162. **(A,B)**: Estimated n-level trajectory using LSTM model. **(C,D)** Prediction vs. observed block accuracy at each block using LSTM model.

The RMSE values are 9.34 and 18.77% for Experiment 1 and Experiment 2, respectively. Studying the predicted N-level trajectories of the chosen samples in Figures 14, 15, we make the following observations. In experiment-1, SP451 is consistently placed at a higher n-level during the oscillations, as such the accuracy values are also wrongly predicted. SP259 and SP438 from the same experiment show highly unstable predicted N-level trajectories, specifically between adjacent levels. This behavior of the model where it blindly follows presented n-backs instead of correctly predicting the true N-level becomes visibly prominent in the trajectory of RLB102 (playing *mini-block moderate*) from experiment 2 (see Figure 15A). Even though the RMSE values are comparable to the HMM and the UKF models, this characteristic wherein the predicted N-level skill follows the presented n-back levels is not always desirable, as seen from the trajectories in Figures 14A–C, 15A,B. We therefore reiterate that a deep-learning framework is not suitable for our application, with the given amount of data. In the future, with increasing datastreams, we can experiment with larger LSTM networks (more parameters) that will not be prone to over-fitting, as it is in our case.

## 5. Discussion

In this paper, multiple ML approaches are presented as a potentially more optimal method of modeling temporal dependencies in the context of adaptive training of WM as compared to conventional growth modeling. We find that all presented models provide accurate predictions of participants' WM skills. However, the Markov models did a better job (compared to UKF and LSTM models) at predicting accuracies. In comparison to the LSTM, the HMMs have very few parameters, thereby explaining the latter's improved performance. We also note that even though we had access to a relatively large WM training data set, this is small compared to that typically needed to achieve reasonable fits with a LSTM model. Additionally, the method estimates the input-output mapping directly (instead of a model from which the input-output relationship must be inferred) which has the benefit of not solving a proxy problem, but the downside that it often cannot be reused to solve related problems. For this problem, the advantages of the Kalman filter model (adaptive rho value) are outweighed by the advantages of the HMM model (non-linear dynamics). Thus, we can conclude that the HMM model is better for these data (that is, its biases are better aligned with the problem).

A key benefit of these models is that they provide a principled alternative to commonly used adaptive algorithms, such as staircases, which are widely used in cognitive training tasks. The key disadvantage of commonly used approaches is that they rely only upon the most recent performance of participants and thus momentary lapses, or lucky streaks, can place participants at inappropriate challenges. This can lead participants to become bored or frustrated and may explain poor performance seen in participants such as SP259 illustrated in Figure 7. While we have not yet demonstrated that the models fit here fully address these issues, they do all have the benefit that their estimates rely upon a different balance of local and past information regarding the participants' cognitive skills. Another key benefit of these models is that they can be naturally extended to deal with higher dimensional datasets, which is a challenge for standard adaptive approaches. For example, it is common in gamified approaches to vary details of tasks, ranging from navigation challenges, uses of stimulus sets from multiple modalities (for which there may be differences in latent memory skill), or when multiple memory tasks are applied. To date, research is limited regarding the most optimal methods to adapt difficulty across a multidimensional space and ML may provide useful and principled approaches. A natural next step is to have challenges be generated by the filtering models, which we hypothesize would lead to a more optimal training experience for participants, thereby promoting task engagement and learning.

An interesting question is the extent to which these models can inform how we think about learning and cognitive processes. There are increasing examples of how artificial neural networks are being studied to understand how information may be represented in biological neural networks (Kriegeskorte, 2015) as well to understand the mechanisms of neural plasticity related to learning (Wenliang and Seitz, 2018). In the context of WM training, there is currently little understanding of the reasons why one participant will exhibit good task learning, or for that matter transfer of learning, while another participant may not learn. Models like the ones presented here attempt to estimate underlying skills that underlie participant task performance and these in turn may help us understand individual differences. For example, while in the current models the models' estimate can be most directly related to propensity to perform the n-back task, the models are all expandable to estimate other internal variables such as those related to guessing behavior, vigilance, attention span, skill to deal with distractions, learning rates, etc. Further, models that can assign task challenges, rather than just track them, can then also design behavioral challenges explicitly to probe these parameters and differentiate them from each other giving rise to the possibility to use these training programs not only to improve cognitive performance but to continuously estimate varied cognitive proficiency over time (Seitz, 2018).

We note that there are numerous ways to expand upon and improve the models presented here. For example, a consequence of limited availability of data is the degradation in performance for high variance datasets, such as found in Experiment 2. To improve stability, a bagging strategy could be adopted in future work, which is an ensemble combination of models trained on random sub-samples of an initial training set (Breiman, 1996). Also worth noting is, in having universal item-parameters, we assume that all participants possess equal discrimination power, and this could be a source of bias.

## 6. Conclusion

In conclusion, our results suggest the filtering and deep-learning approaches can serve as powerful approaches to estimate participants' underlying skills when performing cognitive tasks. However, future work will be required to test the extent to which challenges put forth by these models provide for better learning on the trained task as compared to standard adaptive approaches, and further transfer to untrained tasks, which is the target of most cognitive training tools. It is difficult to predict what changes a proliferation of data will bring, but these techniques are worth exploring for larger data-streams. We note that there is no universally superior ML algorithm and future research will be required detail the properties of models beyond those presented here.

## Data Availability Statement

The datasets generated for this study are available on request to the corresponding author.

## Ethics Statement

The studies involving human participants were reviewed and approved by University of California, Riverside Human Research Review Board. The patients/participants provided their written informed consent to participate in this study.

## Author Contributions

SS wrote the manuscript and designed and conducted the modeling analyses, and co-designed the models. CS designed the models and co-wrote manuscript. AP designed and ran the experimental studies, processed the resulting data, and co-wrote manuscript. SJ designed and oversaw the experimental studies and assisted with the manuscript. AS designed and oversaw the project and co-wrote the manuscript. All authors contributed to the article and approved the submitted version.

## Conflict of Interest

SJ has an indirect financial interest in the MIND Research Institute, Irvine, CA, whose interests are related to this work. The remaining authors declare that the research was conducted in the absence of any commercial or financial relationships that could be construed as a potential conflict of interest.

## Footnotes

^1^We generally refer to the accuracy as the fraction of correct responses (see Equation 1). However, for ease of notation, for the equations in this section, *y*_*t*_ is the numerator of that expression (the *count of the number* correct) and *m*_*t*_ is the denominator (the total number of targets). *m*_*t*_ is fixed, so a distribution on the count straightforwardly implies a distribution on the fraction.

^2^There are also four other parameters for the top and bottom row of Equation (6). As one cannot transition lower than the top row or higher than the bottom row, we give them their own parameters.

## References

[B1] Au J. Buschkuehl M. Duncan G. J. Jaeggi S. M. (2016). There is no convincing evidence that working memory training is not effective: a reply to melby-lervåg and hulme (2015). Psychon. Bull. Rev. 23, 331–337. 10.3758/s13423-015-0967-4 26518308

[B2] Baddeley A. (2012). Working memory: theories, models, and controversies. Annu. Rev. Psychol. 63, 1–29. 10.1146/annurev-psych-120710-100422 21961947

[B3] Barton M. Lord F. (1981). An upper asymptote for the three-parameter logistic item-response model. ETS Res. Rep. Ser. 1981, i-8. 10.1002/j.2333-8504.1981.tb01255.x26400070

[B4] Baum L. E. Petrie T. Soules G. Weiss N. R. (1970). A maximization technique occurring in the statistical analysis of probabilistic functions of Markov chains. Ann. Math. Stat. 41, 164–171. 10.1214/aoms/1177697196

[B5] Bengio Y. Frasconi P. (1994). An input output hmm architecture, in Proceedings of the 7th International Conference on Neural Information Processing Systems, NIPS'94 (Cambridge, MA: MIT Press), 427–434.

[B6] Breiman L. (1996). Bagging predictors. Mach. Learn. 24, 123–140. 10.1007/BF00058655

[B7] Chan K. Lenard C. Mills T. (2012). An introduction to Markov chains, in The Forty-Nineth Annual Conference of the Mathematical Associatoin of Victoria (Bundoora, VIC).

[B8] Dempster A. P. Laird N. M. Rubin D. B. (1977). Maximum likelihood from incomplete data via the em algorithm. J. R. Stat. Soc. Ser. B 39, 1–38. 10.1111/j.2517-6161.1977.tb01600.x

[B9] Deveau J. Jaeggi S. M. Zordan V. Phung C. Seitz A. R. (2015). How to build better memory training games. Front. Syst. Neurosci. 8:243. 10.3389/fnsys.2014.00243 25620916 PMC4288240

[B10] Green C. Bavelier D. Kramer A. Vinogradov S. Ansorge U. Ball K. (2018). Improving methodological standards in behavioral interventions for cognitive enhancement. J. Cogn. Enhance. 3, 2–29. 10.1007/s41465-018-0115-y

[B11] Hambleton R. K. Swaminathan H. (1984). Item Response Theory: Principles and Applications. New York, NY: Springer. 10.1007/978-94-017-1988-9

[B12] Hochreiter S. Schmidhuber J. (1997). Long short-term memory. Neural Comput. 9, 1735–1780. 10.1162/neco.1997.9.8.1735 9377276

[B13] Julier S. J. Uhlmann J. K. (1997). New extension of the Kalman filter to nonlinear systems, in SPIE 3068: Signal Processing, Sensor Fusion, and Target Recognition VI (San Diego, CA). 10.1117/12.280797

[B14] Karbach J. Unger K. (2014). Executive control training from middle childhood to adolescence. Front. Psychol. 5:390. 10.3389/fpsyg.2014.00390 24847294 PMC4019883

[B15] Kriegeskorte N. (2015). Deep neural networks: a new framework for modeling biological vision and brain information processing. Annu. Rev. Vis. Sci. 1, 417–446. 10.1146/annurev-vision-082114-035447 28532370

[B16] Martin A. Quinn K. (2002). Dynamic ideal point estimation via Markov chain Monte Carlo for the U.S. supreme court, 1953–1999. Polit. Anal. 10:134 10.1093/pan/10.2.134

[B17] Melby-Lervåg M. Hulme C. (2013). Is working memory training effective? A meta-analytic review. Dev. Psychol. 49, 270–291. 10.1037/a0028228 22612437

[B18] Melby-Lervåg M. Redick T. S. Hulme C. (2016). Working memory training does not improve performance on measures of intelligence or other measures of “far transfer”: evidence from a meta-analytic review. Perspect. Psychol. Sci. 11, 512–534. 10.1177/1745691616635612 27474138 PMC4968033

[B19] Mohammed S. Flores L. Deveau J. Hoffing R. Phung C. Parlett C.. (2017). The benefits and challenges of implementing motivational features to boost cognitive training outcome. J. Cogn. Enhance. 1, 491–507. 10.1007/s41465-017-0047-y 30221244 PMC6136448

[B20] Pedullà L. Brichetto G. Tacchino A. Vassallo C. Zaratin P. Battaglia M.. (2016). Adaptive vs. non-adaptive cognitive training by means of a personalized app: a randomized trial in people with multiple sclerosis. J. Neuroeng. Rehabil. 13:88. 10.1186/s12984-016-0193-y 27716336 PMC5050994

[B21] Pergher V. Shalchy M. A. Pahor A. Hulle M. M. V. Jaeggi S. M. Seitz A. R. (2019). Divergent research methods limit understanding of working memory training. J. Cogn. Enhance. 4, 100–120. 10.1007/s41465-019-00134-7 PMC8336689 34355115

[B22] Rabiner L. R. (1990). A tutorial on hidden markov models and selected applications in speech recognition, in Readings in Speech Recognition, eds Waibel A. Lee K.F. (San Francisco, CA: Morgan Kaufmann Publishers Inc.), 267–296. 10.1016/B978-0-08-051584-7.50027-9

[B23] Rasch G. (1980). Probabilistic Models for Some Intelligence and Attainment Tests. Chicago, IL: University of Chicago Press.

[B24] Rebane J. (2018). Seq 2 Seq RNNs and ARIMA models for cryptocurrency prediction: a comparative study, in Proceedings of the 24th ACM SIGKDD Conference on Knowledge Discovery and Data Mining (London, UK).

[B25] Redick T. S. (2019). The hype cycle of working memory training. Curr. Direct. Psychol. Sci. 28, 423–429. 10.1177/0963721419848668 31814661 PMC6897530

[B26] Rutledge K. van den Bos W. McClure S. M. Schweitzer J. (2012). Training cognition in ADHD: Current findings, borrowed concepts, and future directions. Neurotherapeutics 9, 542–558. 10.1007/s13311-012-0134-9 22911054 PMC3441933

[B27] Särkkä S. (2013). Bayesian Filtering and Smoothing. New York, NY: Cambridge University Press.

[B28] Seitz A. R. (2018). A new framework of design and continuous evaluation to improve brain training. J. Cogn. Enhance. 2, 78–87. 10.1007/s41465-017-0058-8 29868648 PMC5984043

[B29] Simons D. Boot W. Charness N. Gathercole S. Chabris C. Hambrick D.. (2016). Do brain-training programs work? Psychol. Sci. Publ. Interest 17, 103–186. 10.1177/1529100616661983 27697851

[B30] Soveri A. Antfolk J. Karlsson L. Salo B. Laine M. (2017). Working memory training revisited: a multi-level meta-analysis of n-back training studies. Psychon. Bull. Rev. 24, 1077–1096. 10.3758/s13423-016-1217-0 28116702

[B31] Soveri A. Antfolk J. Karlsson L. Salo B. Laine M. (2018). Working memory training revisited: a multi-level meta-analysis of n-back training studies. Psychon. Bull. Rev. 10.31234/osf.io/fvyra 28116702

[B32] Stepankova Georgi H. Lukavsky J. Buschkuehl M. Kopecek M. Ripova D. Jaeggi S. (2013). The malleability of working memory and visuospatial skills: a randomized controlled study in older adults. Dev. Psychol. 50, 1049–1059. 10.1037/a0034913 24219314

[B33] Valsiner J. Molenaar P. Lyra M. Chaudhary N. (2009). Dynamic Process Methodology in the Social and Developmental Sciences. Dordrecht: Springer 10.1007/978-0-387-95922-1

[B34] Wang X. Berger J. Burdick D. (2013). Bayesian analysis of dynamic item response models in educational testing. Ann. Appl. Stat. 7, 126–153. 10.1214/12-AOAS608

[B35] Wenliang L. Seitz A. (2018). Deep neural networks for modeling visual perceptual learning. J. Neurosci. 38, 1620–1627. 10.1523/JNEUROSCI.1620-17.2018 29793979 PMC6031581

