# Supplementary material for: Application of Machine Learning Models for Tracking Participant Skills in Cognitive Training
Source: Front Psychol. 2020 Jul 22;11:1532. doi: 10.3389/fpsyg.2020.01532 (PMC7387708; doi:10.3389/fpsyg.2020.01532)
Supplement: Supplementary file 1 [file Data_Sheet_1.pdf]

Application of Machine Learning Models for Tracking Participant Skills in Cognitive Training

1 SUPPLEMENTARY FIGURES

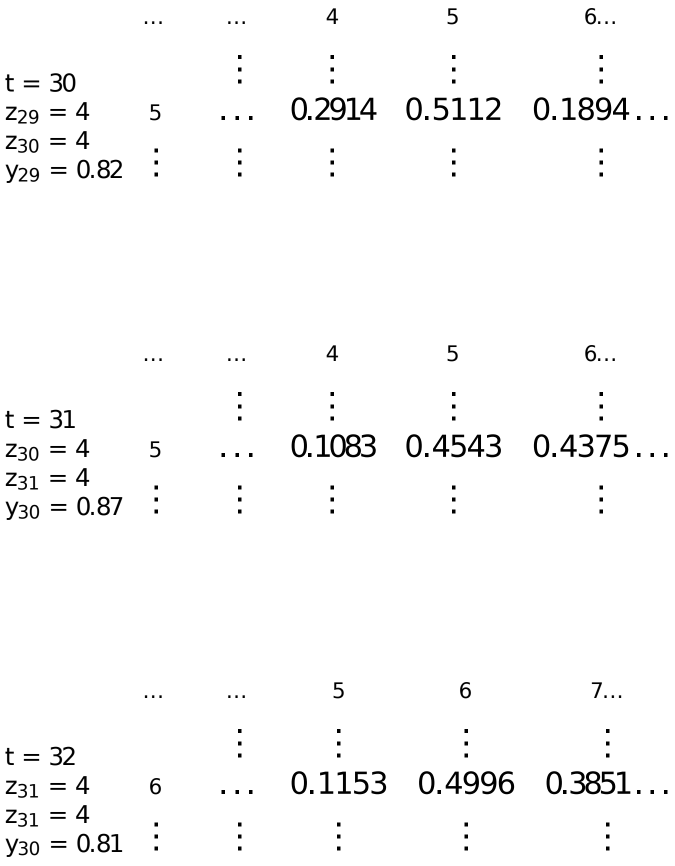

Figure S1. The evolving transition structure in Model-2 for participant SP438 from experiment 1. Transitions in (top matrix) show values before promotion where probability from 5 to 6 is low, (middle matrix) indicate a higher tendency to move from 5 to 6 (here's when promotion occurs) and (bottom matrix) indicate high tendency to remain at 6 and low tendency to go back to 5, i.e; values after promotion.

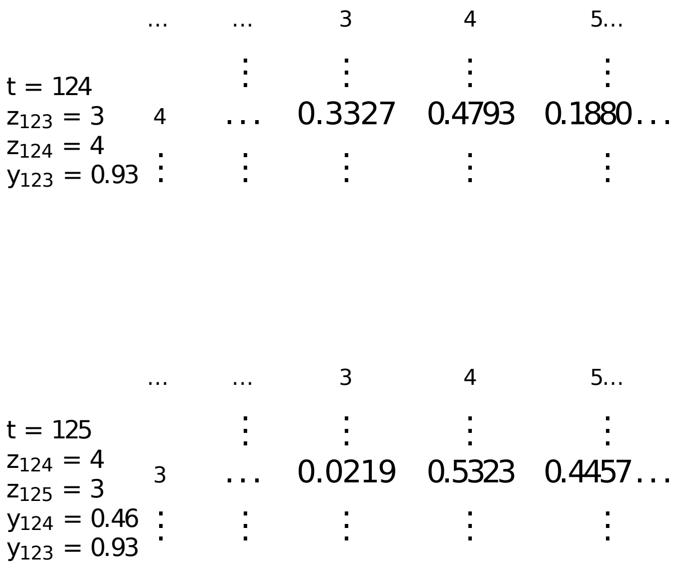

Figure S2. The evolving transition structure in Model-2 for participant SP259 from experiment 1. On comparing transition from n-level 5 to n-level 4 at t=124 and t=125, we note that the probability is slightly increased, and the accuracy drops to 0.46, leading to demotion.

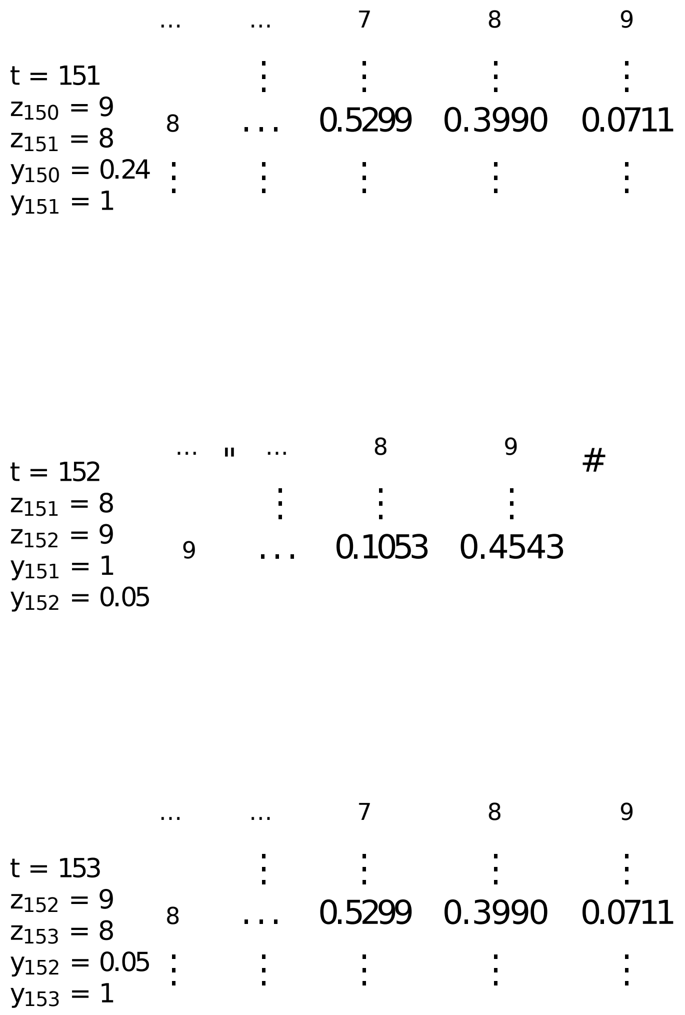

Figure S3. The evolving transition structure in Model-2 for participant SP451 from experiment 1. The transitions have higher tendencies to remain in the same state for the whole sequence, however the alternating rise and fall of accuracies pushes states back and forth between 8 and 9.

… 4 5 6…

t = 128
z_{127} = 5
z_{128} = 5
y_{127} = 0.625
y_{128} = 0.667

5 … 0.5079 0.0175 0.4746…

… … 5 6 7…

t = 129
z_{129} = 6
z_{128} = 5
y_{128} = 0.667
y_{129} = 0.667

6… … 0.5348 0.0031 0.4621…

… … 6 7 8…

t = 130
z_{129} = 6
z_{130} = 7
y_{130} = 0.667
y_{129} = 0.667

7… … 0.5348 0.0031 0.4621…

Figure S4. The evolving transition structure in Model-2 for participant RLB162 from experiment 2. The transitions show high probabilities to move from current to the next level. This is coupled with high accuracy, that pushes the participant to the next consecutive n-level.

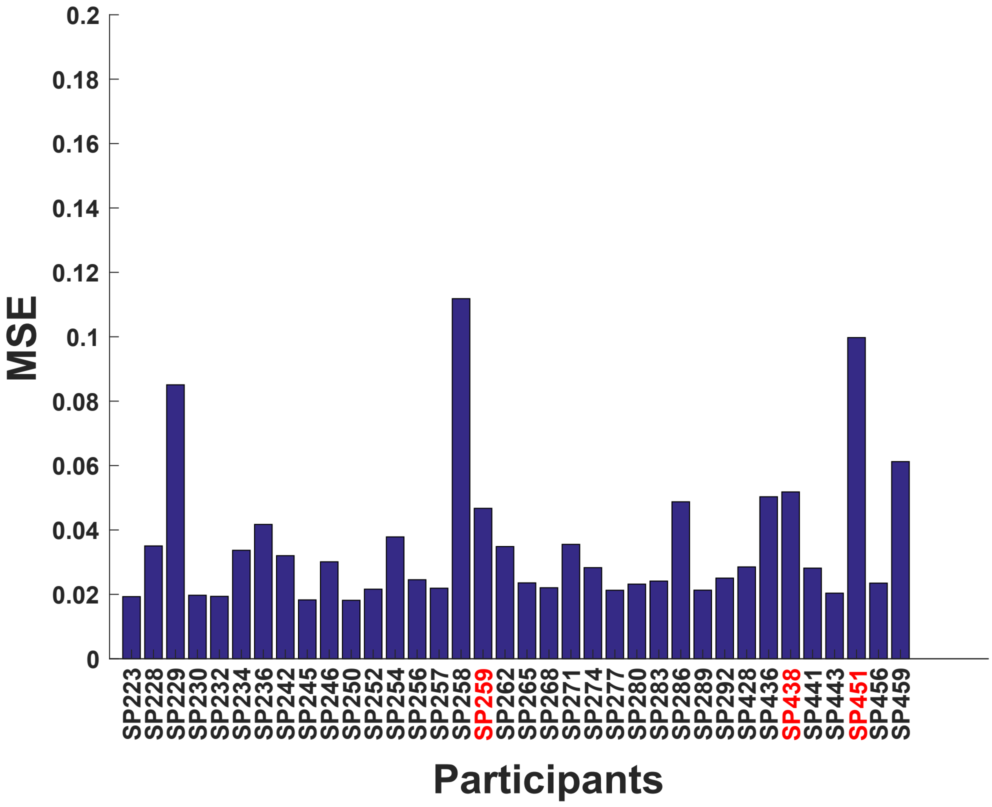

Figure S5. MSE across all participants for the UKF model for experiment 1. The participants highlighted in red correspond to the samples picked below for analysis.

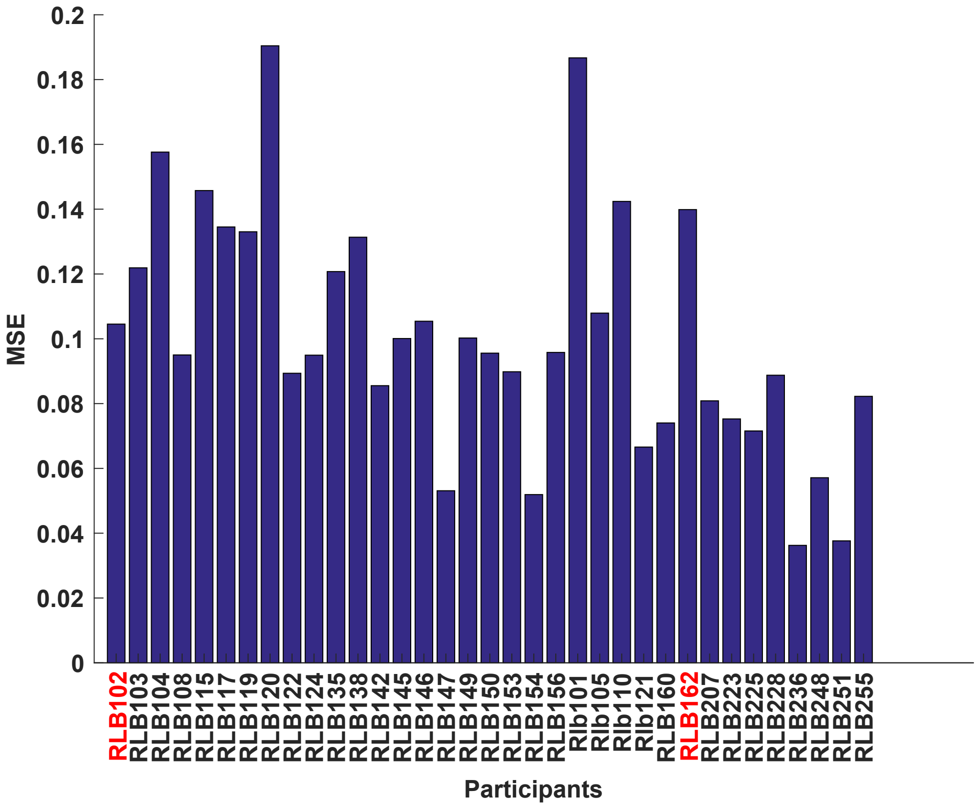

Figure S6. MSE across all participants for the UKF model for experiment 2 .The participants highlighted in red correspond to the samples picked below for analysis.

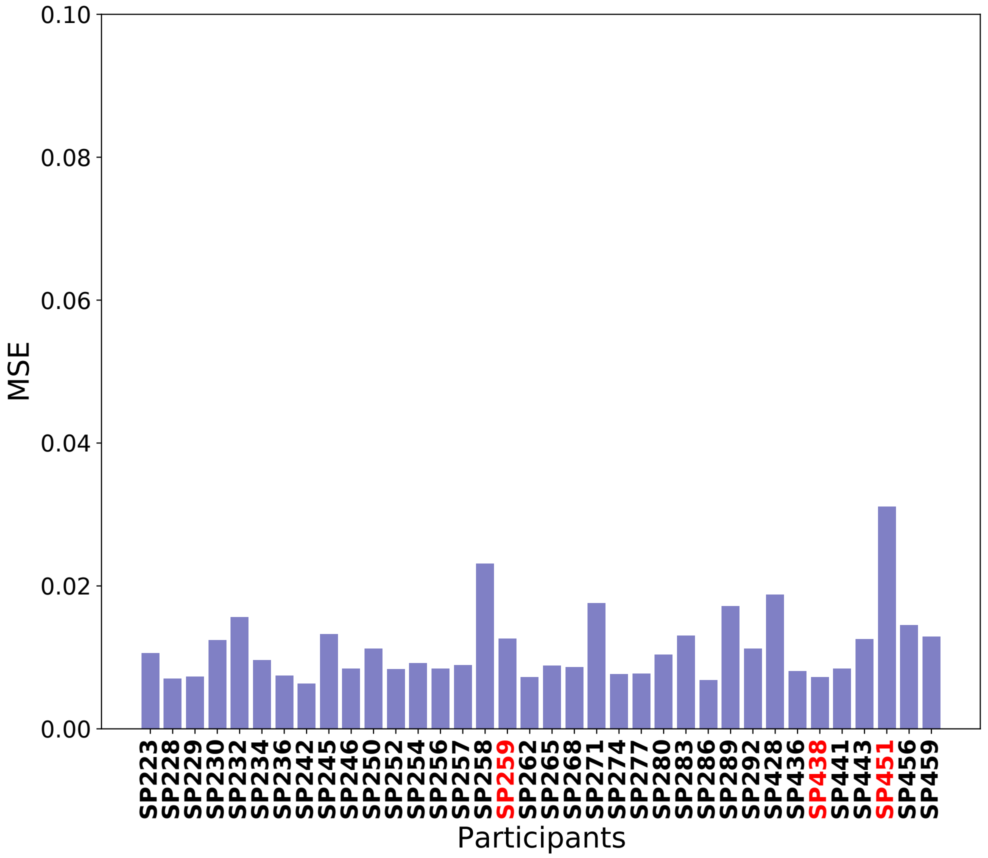

Figure S7. MSE across all participants for the LSTM model for experiment 1. The participants highlighted in red correspond to the samples picked below for analysis.

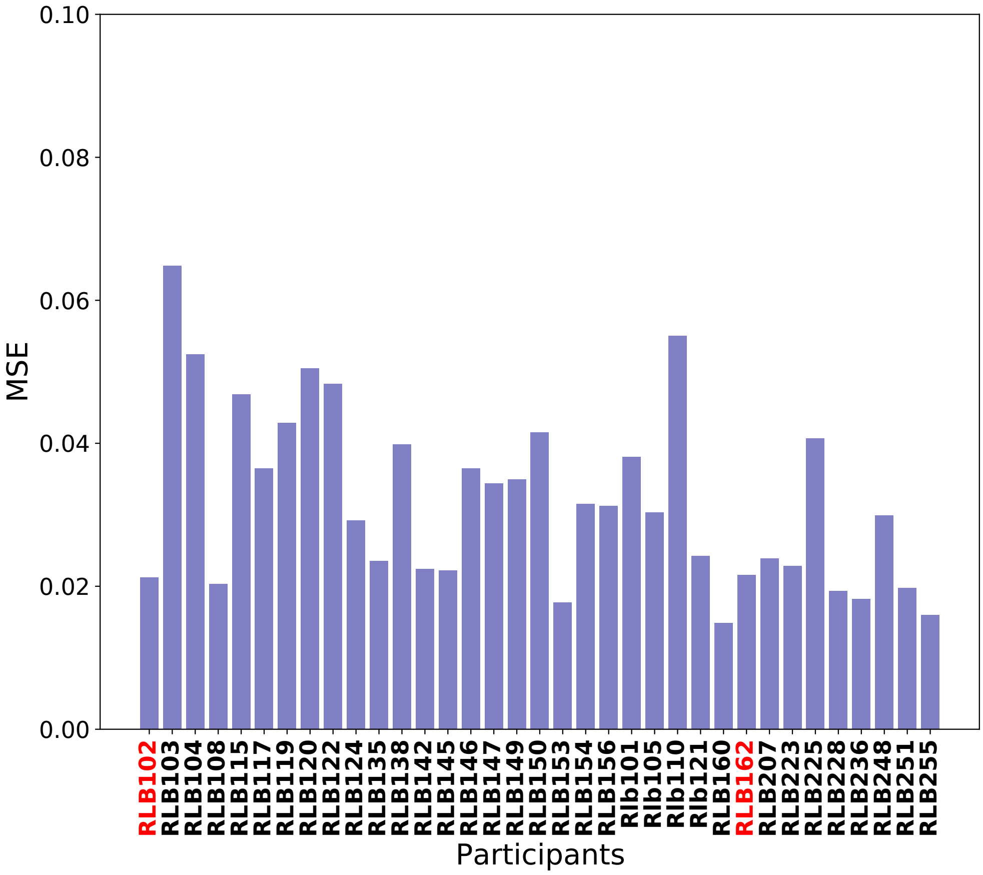

Figure S8. MSE across all participants for the LSTM model for experiment 2. The participants highlighted in red correspond to the samples picked below for analysis.
